# Body shape indices are predictors for estimating fat-free mass in male athletes

**DOI:** 10.1371/journal.pone.0189836

**Published:** 2018-01-18

**Authors:** Yohei Takai, Miyuki Nakatani, Toru Aoki, Daisuke Komori, Kazuyuki Oyamada, Kensuke Murata, Eiji Fujita, Takuya Akamine, Yoshihisa Urita, Masayoshi Yamamoto, Hiroaki Kanehisa

**Affiliations:** National Institute of Fitness and Sports in Kanoya, Kanoya, Japan; The University of Tokyo, JAPAN

## Abstract

It is unknown whether body size and body shape parameters can be predictors for estimating whole body fat-free mass (FFM) in male athletes. This study aimed to investigate whether body size and shape variables can be predictors for FFM in male athletes. Using a whole-body dual-energy X-ray absorptiometry scanner, whole body fat mass (FM) and FFM were determined in 132 male athletes and 14 sedentary males. The sample was divided into two groups: validation (N = 98) and cross-validation (N = 48) groups. Body height (BH), body mass (BM), and waist circumference at immediately above the iliac crest (W) were measured. BM-to-W and W-to-BH ratios were calculated as indices of body shapes. Stepwise multiple regression analysis revealed that BM/W and W/BH were selected as explainable variables for predicting FFM. The equation developed in the validation group was FFM (kg) = 0.883 × BM/W (kg/m) + 43.674 × W/BH (cm/cm)– 41.480 [*R*^2^ = 0.900, SEE (%SEE) = 2.3 kg (3.8%)], which was validated in the cross-validation group. Thus, the current results demonstrate that an equation using BM/W and W/BH as independent variables is applicable for predicting FFM in male athletes.

## Introduction

Major components of body composition are adipose, skeletal muscle, bone, visceral, and brain tissues. In particular, whole body fat-free mass (FFM) is of interest in the field of sports science as a component of body composition and has been found to be associated with talent identification, athletic performance, and body mass management. In fact, prospective college male soccer players have greater FFM relative to body height squared [1]. In addition, FFM is strongly related to one repetition maximum load during squat, deadlift, and bench press [2], indicating that it is a potential indicator of force-generating capacity. Furthermore, FFM is often utilized to estimate resting energy expenditure [3]. From the viewpoint of physical resources, assessment of FFM may provide useful information for sports scientists and coaches to improve body composition for designing training regimens and managing body mass.

FFM is extensively used to determine dual-energy x-ray absorptiometry (DXA) in various research fields. FFM determined by DXA techniques is highly correlated with total body skeletal muscle mass measured as measured by magnetic resonance imaging [4]. In addition, DXA-based determinations of whole-body and segment FFMs in athletes have been found to have good accuracy and reliability [5]. However, the device is expensive and is not portable, thus this technique has limitations for application in field survey sampling in large populations. As one alternative approach, anthropometric prediction models have been developed for various populations (e.g. children, the elderly and athletes) [6–8]. Prediction models use the circumferences of arm, thigh, calf, skinfold, and body height as independent variables. The accuracy of the developed equations for predicting FFM have previously been established [7]. In addition, anthropometric variables derived from body height and mass are associated with FFM in athletes [1]. Furthermore, body mass-to-waist ratio (BM/W) has been shown to be a strong indicator for whole body skeletal muscle volume in children [9]. In previous studies, however, the validity of the prediction equations derived from these variables have not been tested. Body height and mass and waist circumference are conveniently determined without a special measurement technique or apparatus. For athletes, if body size and shape indices derived from these anthropometric variables can be predictors for FFM, it could be a more convenient measure for estimating FFM in comparison to traditional anthropometric prediction models.

Proportions of segment fat mass (FM) and FFM to total FM and FFM within the whole body for athletes is event-specific, owing to different movements and energy requirements or habitual training regimens [10–12], and is different for trained versus untrained men [13]. For example, the muscle size of wrist flexors is larger in weight lifters than in wrestlers, whereas FFM is the same between the two groups [11]. Furthermore, it has been shown that a breakpoint exists in the relationship between FFM and body mass in NFL football players [14], which implies that the accumulation of FM and FFM relative to body mass may be different above and below the breakpoint. These prior findings indicate that the proportion of FM and FFM to body mass differ according to the types of competitive sports, the presence or lack of habitual training and the magnitude of body mass. Thus, one can assume that, for athletes, the accuracy of FFM estimated from a prediction equation in which body size and shape are independent variables will be influenced by the proportion of segment FM and FFM to total FM and FFM within the whole body. This study sought to elucidate whether body size and shape variables can be predictors of FFM in order to develop a prediction equation for estimating FFM in male athletes.

## Materials and methods

### Subjects

A total of 146 male athletes and sedentary males (21.2 ± 2.0 yrs; 172.5 ± 6.6 cm; 74.9 ± 15.6 kg) voluntarily participated in this study. The inclusion criterion for the athletes was current involvement in competitive sports at national and international levels. The investigation was conducted during in-season for all subjects. The sample of the athletes was consisted of soccer players (N = 32), cyclists (N = 9), jumpers (N = 13), shot put and javelin throwers (N = 13), gymnasts (N = 16), Judo (N = 37) and Kendo (N = 12) athletes. They took part in regular event-specific training for more than five days (>1.5 hours/day) per week, and for at least five years. The sedentary males (N = 14) did not participated in regular physical training. They were free of cardiovascular, metabolic, and immunologic disorders and/or orthopedic abnormalities, and were not using any medications that affected their muscle function. This investigation was conducted according to the Declaration of Helsinki, and was approved by the National Institute of Fitness and Sports in Kanoya's Ethics Committee for human experimentation. Prior to the experiment, all subjects were informed of the experimental procedures of this study and possible risks of the measurements beforehand. Written informed consent was obtained from each subject.

### Measurements of anthropometry

Body height (BH) and mass (BM) were measured using a stadiometer and a leg-to-leg bioelectrical impedance analyzer with a computer-programmed athletic mode (DC-320, TANITA, Japan) to the nearest 0.1 cm and 0.1 kg, respectively. Participants were instructed to restrain from alcohol intake for 24 h prior to the experiment and from having a meal 2 h prior to the measurement. Waist circumference (W) was measured to the nearest 0.1 cm with a cloth tape (Rotary measure, Sodeyama Co., Ltd. Japan) immediately above the iliac crest [15]. To confirm the reproducibility of the waist circumference measurement, we measured twice at least three days apart for nine male athletes. The intra-class correlation coefficient (ICC) for the measurement was 0.97. The measurement error between 1st and 2nd measurements was 0.0 ± 1.1 cm, and the coefficient of variance (CV) was 0.9 ± 0.6%.

### Measurement of body composition

Percent fat mass (%FM), whole body and segmental body composition were measured using a whole-body dual-energy X-ray absorptiometry (DXA) scanner (Hologic Delphi A-QDR, USA). Participants lay supine on a bed with arms and legs straight. From the obtained radiography, we divided the body into three segments: trunk, upper, and lower extremities with built-in software (Hologic Delphi A-QDR, USA) and estimated whole body and segmental fat mass (FM) and FFMs. Room temperature was usually kept at 22°C. DXA-derived body composition has been shown to have good accuracy and reliability in sport athletes [5]. To confirm the reproducibility of the DXA measurement, we measured body composition twice at least three days apart for nine male athletes. The ICCs were 0.92 for FM and 0.91 for FFM, respectively. The measurement errors between 1st and 2nd measurements were -0.6 ± 0.6 kg for FM and 0.3 ± 2.0 kg for FFM, and the CVs were 5.4 ± 4.5% for FM and 1.8 ± 1.4% for FFM, respectively.

### Data analysis

Body mass-to-waist circumference ratio (BM/W) was calculated from BM and W [9], and waist circumference-to-body height ratio (W/BH) was calculated based upon BH and W [16]. Since body shape parameters have been found to be correlated with FFM in male athletes [1] and are indicators of prospective male athletes [17], body mass index (BMI) (kg/m^2^) and reciprocal ponderal index (RPI) (cm/kg^0.333^) were calculated based upon BH and BM.

**Fig 1:**
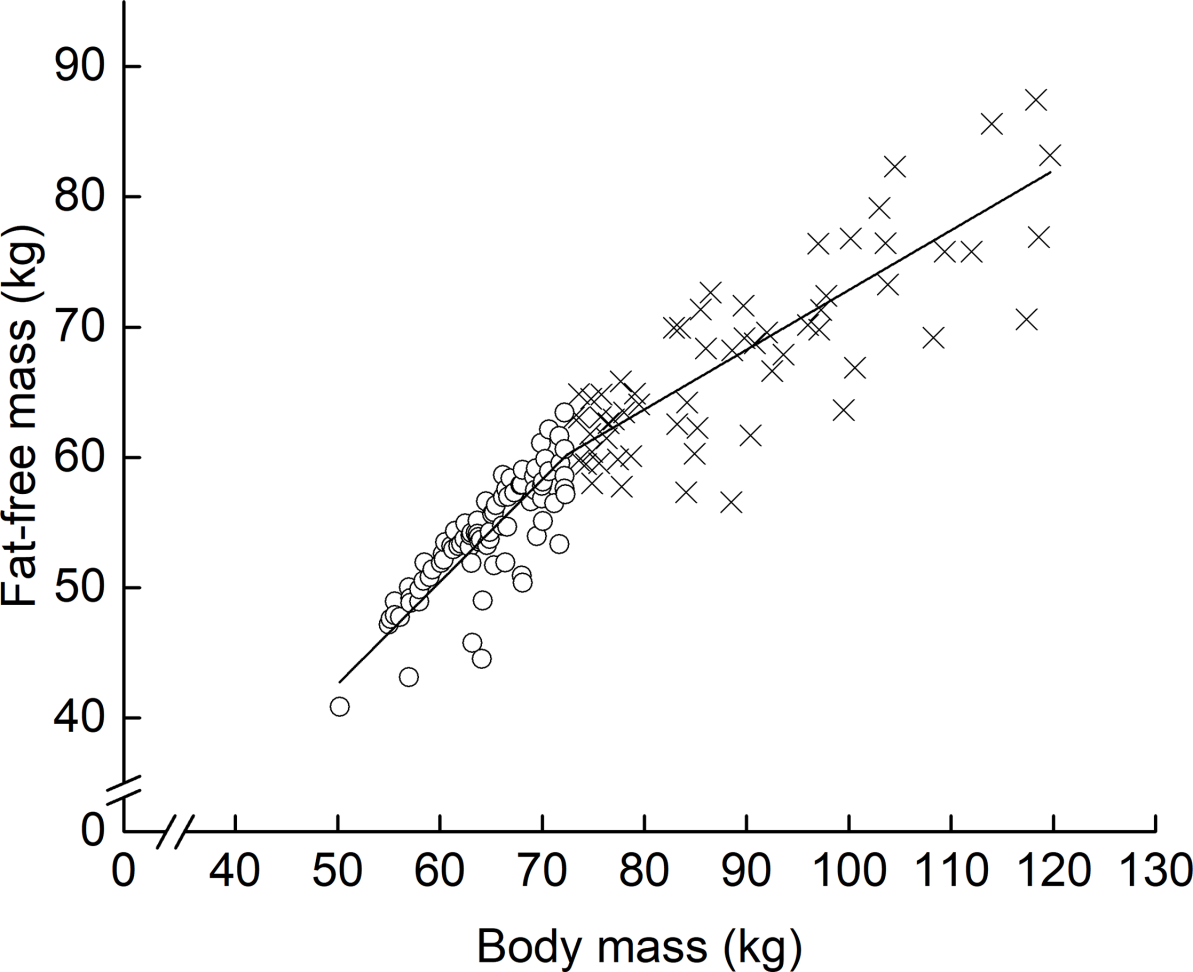
Relationship between fat-free mass and body mass. Open circle, subjects with below 72.4 kg of BM; Open triangle, subjects with above 72.4 kg of BM.

To examine the event-specific differences in the ratio of segment FM and FFM to whole-body ones, respectively, each FM and FFM segment was divided by whole-body FM and FFM, respectively. Furthermore, to detect a breakpoint in the relationship between measured FFM and BM, piecewise linear regression analysis was conducted across all subjects. As the result, there was a breakpoint (72.4 kg of BM) in the corresponding relationship (Fig 1). This finding indicates that the proportion of FM and FFM to BM differs above and below the breakpoint.

To develop the prediction equation for whole body FFM, participants were randomly assigned to either a validation (n = 98) or a cross-validation (n = 48) group (Table 1) without significant differences in all measured variables. A random function from the spreadsheet software (Microsoft Excel 2010, Microsoft Co., Washington, USA) was used to assign participants to the validation or cross-validation group. A prediction equation was developed by applying a stepwise multiple regression analysis to the validation group. After the developed equation was validated, this equation was applied to the cross-validation group. In the two analyses, the residual error was calculated as the difference between the measured and estimated FFM values (measured FFM–estimated FFM).

**Table 1:** Physical characteristics of validation and cross-validation groups.

	Validation (N = 98)			Cross-validation (N = 48)		
Age, yrs	21.3	±	2.1	21.0	±	1.9
Body height, cm	172.5	±	6.9	172.4	±	5.9
Body mass, kg	75.0	±	16.1	74.8	±	14.6
BMI, kg/m^2^	25.0	±	4.3	25.1	±	4.4
RPI, m/kg^0.333^	41.2	±	2.0	41.2	±	2.2
Waist circumference, cm	81.4	±	11.2	81.0	±	10.4
Waist-to-body height ratio, cm/cm	0.5	±	0.1	0.5	±	0.1
Body mass-to-waist ratio, kg/m	91.3	±	8.2	91.7	±	7.3
%FM, %	14.1	±	7.0	13.9	±	5.9
FM, kg	11.1	±	7.8	10.9	±	6.8
FFM, kg	59.7	±	9.3	59.8	±	7.9
Number of subjects in each event (% of each event to total number within each group)						
Control	10 (10%)			4 (8%)		
Soccer players	23 (23%)			9 (19%)		
Cyclist	7 (7%)			2 (4%)		
Jumper	6 (6%)			7 (15%)		
Kendo athletes	8 (8%)			4 (8%)		
Judo athletes	24 (24%)			13 (27%)		
Gymnast	11 (11%)			5 (10%)		
Thrower	9 (9%)			4 (8%)		
Number of above and below a breakpoint of body mass (% of above or below a breakpoint to total number within each group)						
above 72.4 kg of body mass	59 (60%)			26 (54%)		
below 72.4 kg of body mass	39 (40%)			22 (46%)		

Values are expressed as means and SDs.

BMI, body mass index; RPI, reciprocal ponderal index; %FM, DXA-derived percent fat mass; FM, DXA-derived fat mass; FFM, DXA-derived fat-free mass

### Statistical analysis

Descriptive data are presented as means ± SDs. An unpaired Student t-test was used to test differences in the independent variables between validation and cross-validation groups. For all participants, Pearson’s product-moment correlation analysis was performed to determine associations between DXA-derived variables and other anthropometric variables. Correlation coefficients were interpreted as being trivial (*r*<0.1), small (0.1<*r*<0.3), moderate (0.3<*r*<0.5), and large (0.5<*r*<0.7), very large (0.7<*r*<0.9), nearly perfect (*r*>0.9) or perfect (*r* = 1) according to Hopkins [18]. Two way analysis of variance (ANOVA) with repeated measures was used to detect significant event-related differences in the proportion of segment FFM to whole-body FFM. When the significant interactions between event and segment were found, simple main effect tests were conducted for post hoc comparisons.

To develop the prediction equation, a stepwise multiple regression analysis was first conducted with FFM as the dependent variable and two dummy variables (athletes or sedentary = 0 or 1; above or below 72.4 kg of BM = 0 or 1), anthropometric variables and body shape indices (BH, BM, BMI, RPI, W, W/BH, BM/W) as independent variables. We adopted the explainable variables which satisfied with the conditions that the variance inflation factor and condition indicators were < 10 and < 30, respectively, to avoid multicollinearity of the prediction equation. Second, we examined whether the regression slope and intercept in the relationship between the measured and estimated FFM values significantly differed from 1 and 0, respectively. Third, the residual of the estimate was plotted against the mean values of the measured and estimated FFM to confirm the systematic error, as described by Bland and Altman [19]. The three tests described above were used to examine the validity of the developed prediction equation. Once the prediction equation developed in the validation group was validated, it was applied to the cross-validation group, and we confirmed whether or not the aforementioned three criteria were satisfied. The standard error of estimate (SEE) was calculated to evaluate the accuracy of the estimate obtained by the developed prediction equation. The SEE was expressed as both an absolute value and relative to the mean value of the measured FFM. One-way ANOVA was used to detect the event-related differences in SEEs. Pearson’s product-moment correlation (*r*) analysis was conducted to examine the influence of the distribution of FM and FFM within the whole body on SEEs. The probability level for all statistical analysis was set at *p*<0.05. Data were analysed using a statistical software program (SPSS statistics 22.0, IBM Co., New York, USA).

## Results

### Event-related differences in DXA-derived %FM, FM and FFM

**Fig 2:**
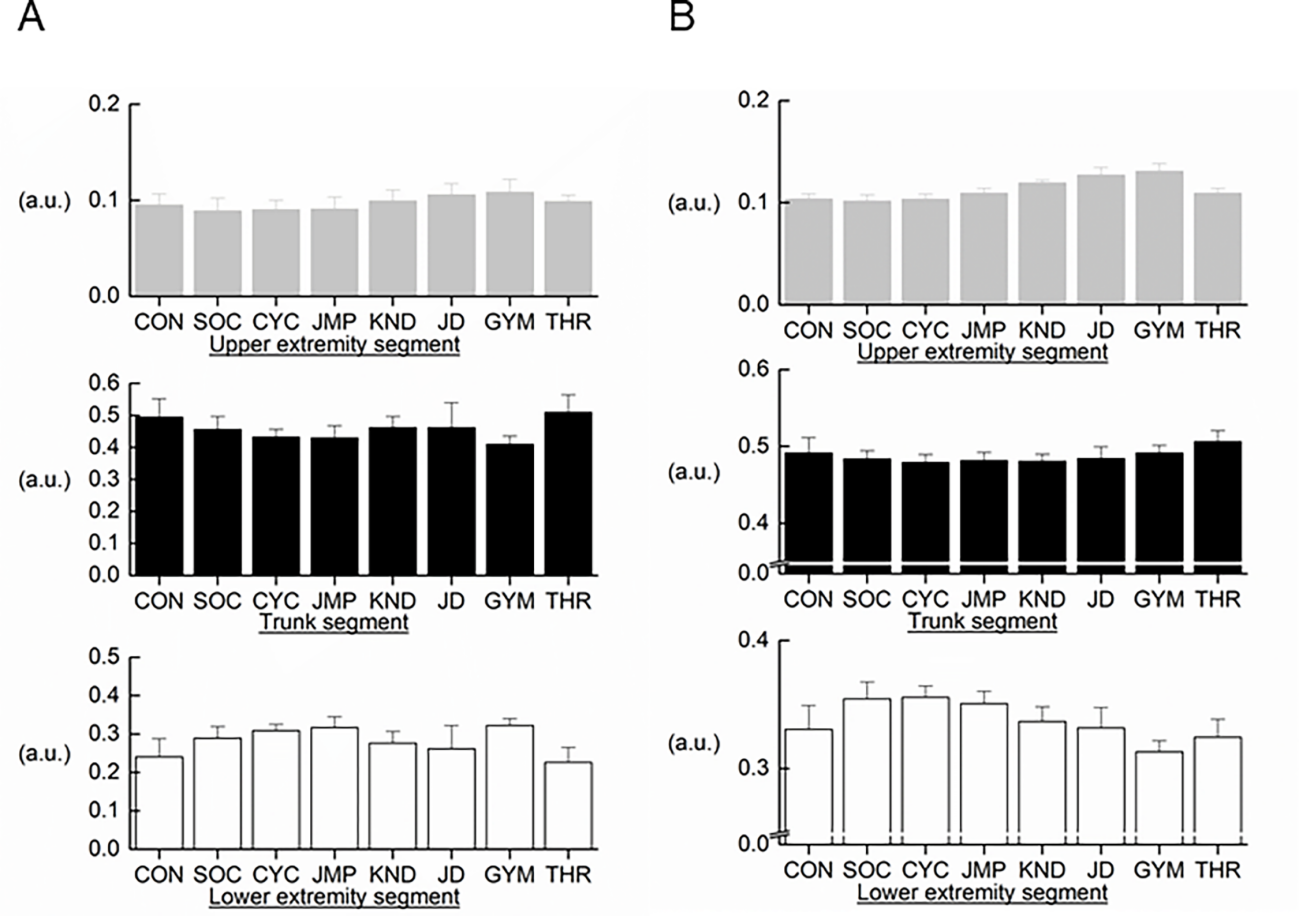
**Proportion of segment body composition to whole body composition in fat mass (FM) (A) and fat-free mass (FFM) (B).** CON, sedentary males; SOC, soccer athletes; CYC, cyclists; JMP, jumpers; KND, kendo athletes; JD, judo athletes; GYM, gymnasts; THR, throwers.

ANOVAs revealed that significant interactions between segment and event were found for FM (*F* = 7.718, *d*f = 8.041, *η*^*2*^ = 0.281, p<0.001) and FFM (*F* = 19.601, *d*f = 8.736, *η*^*2*^ = 0.499, p<0.001) (Fig 2). The ratio for the upper extremity segment was greater in gymnasts and Judo athletes relative to soccer players, jumpers, and cyclists. For trunk segments, throwers had the greater ratio in comparison to jumpers, gymnasts, and cyclists. Judo athletes and sedentary men had the greater ratio of trunk segments to whole-body FM than gymnasts. The ratio of the lower extremity segment FM to whole-body FM was lower in throwers and sedentary men than in soccer players, jumpers, gymnasts, and cyclists, and in Judo athletes relative to jumpers and gymnasts.

The ratio of the upper extremity segment was greater in gymnasts and Judo athletes relative to other groups, and in Kendo athletes relative to other groups, with the exception of gymnasts and Judo athletes. Jumpers and throwers exhibited the greater ratio of upper extremity segment FFM to whole-body FFM in comparison to soccer players. For trunk segments, throwers exhibited a greater ratio than other groups, with the exception of gymnasts and sedentary men. The ratio of the lower extremity segment FFM to whole-body FFM was greater in soccer players, jumpers, and cyclists in comparison to other groups, and lower in gymnasts relative to other groups, with the exception of throwers. There was no significant difference in the ratio between Kendo athletes and jumpers.

### Developed equation for estimating whole body FFM

**Fig 3:**
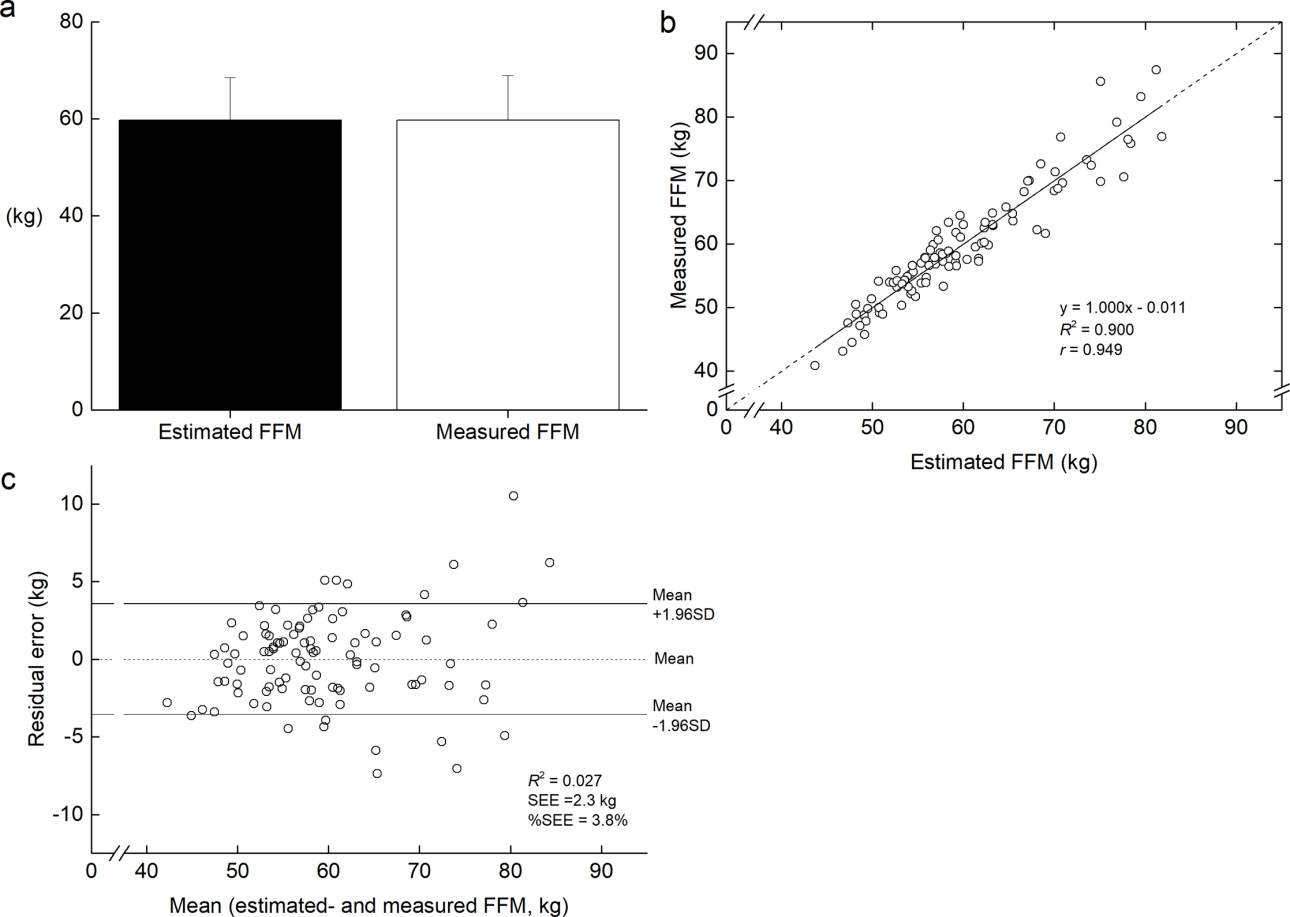
**Comparison the estimated whole-body fat-free mass (FFM) to the measured FFM in validation group (a), and regression between the measured FFM and the estimated FFM (b), and residual vs. the mean values of the measured and estimated FFM values (c).** In panel b, dotted line shows identical line. In panel c, dashed and solid lines indicate the mean values of residual errors and mean ± 1.96 SD, respectively.

In the validation group, whole body %FM, FM and FFM were significantly related to anthropometric variables (p<0.001, Table 2). Among all the variables, the correlation coefficient between FFM and BM/W was the highest (r = 0.920). Stepwise multiple regression analysis revealed that BM/W and W/BH were selected as explainable variables for predicting whole-body FFM. The developed equation was whole-body FFM (kg) = 0.883 × BM/W (kg/m) + 43.674 × W/BH (cm/cm)– 41.480 (*R*^2^ = 0.900, SEE (%SEE) = 2.3 kg (3.8%)). Confidential interval of the coefficient was 0.797 to 0.969 for BM/W, 31.439 to 55.908 for W/BH, and -48.384 to -34.576 for constant, respectively. The two dummy variables were not selected in the prediction of FFM. Estimated FFM did not differ from FFM (Fig 3A). The slope and intercept of the regression line between estimated and measured FFM did not differ from 1 and 0, respectively (Fig 3B). As shown in the Bland-Altman plot (Fig 3C), there was not systematic error.

**Table 2:** Correlation coefficients between DXA-derived variables and anthropometric variables in validation group.

	FM	%FM	FFM
Body height	0.345	0.402	0.592
Body mass	0.748	0.879	0.920
BMI	0.736	0.862	0.835
RPI	-0.661	-0.772	-0.717
Waist circumference	0.839	0.930	0.797
Waist-to-body height ratio	0.816	0.896	0.688
Body mass-to-waist circumference	0.452	0.604	0.919

BH, body height; BM, body mass; W, waist circumference; BMI, body mass index; RPI, reciprocal ponderal index; %FM, DXA-derived percent fat mass; FM, DXA-derived fat mass; FFM, DXA-derived fat-free mass

**Fig 4:**
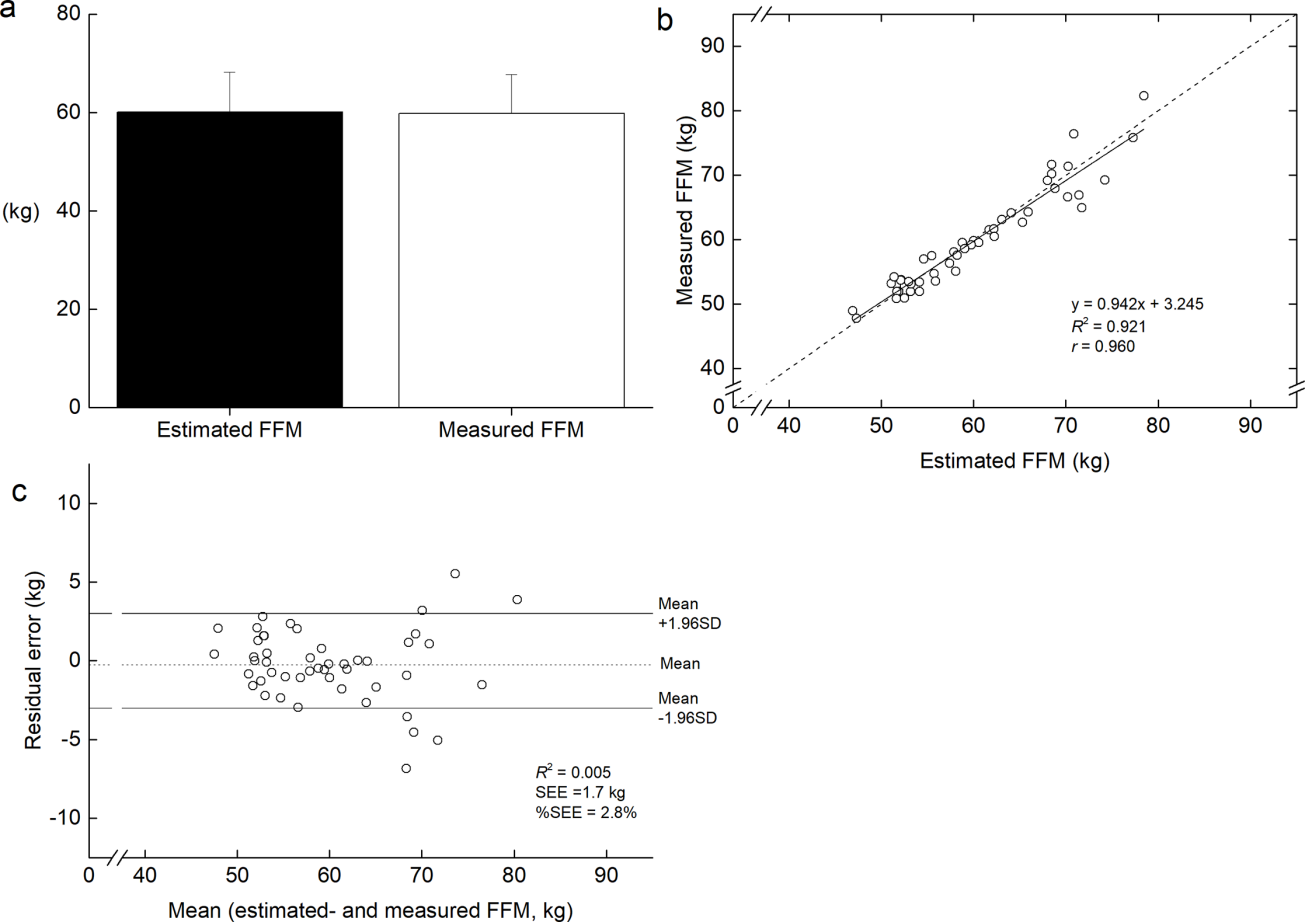
**Comparison the estimated whole-body fat-free mass (FFM) to the measured FFM in cross-validation group (a), and regression between the measured FFM and the estimated FFM (b), and residual vs. the mean values of the measured and estimated FFM values (c).** In panel b, dotted line shows identical line. In panel c, dashed and solid lines indicate the mean values of residual errors and mean ± 1.96 SD, respectively.

In the cross-validation group, the application of the prediction equation developed in the validation group did not produce a significant difference between estimated- and measured FFM (Fig 4A). The slope and intercept of the regression line between estimated- and measured FFM did not differ from 1 and 0, respectively (Fig 4B). The Bland-Altman plot (Fig 4C) revealed that systematic error was not found. The SEE (%SEE) was 1.7 kg (2.8%).

### Relationships between residual errors and the measured variables across all subjects

The values of residual errors were positively related to the measured whole-body FFM (*r* = 0.257, *p*<0.05) and the proportion of segment FFM to whole-body FFM (*r* = 0.305–0.476, *p*<0.05), but was not associated with corresponding variables in FM. There were no significant differences between measured- and estimated whole-body FFM regardless of all event-related groups except for throwers (67.6 ± 1.8 kg vs. 69.1 ± 1.7 kg, -1.5 kg, p<0.05) and sedentary males (51.9 ± 1.7 kg vs. 54.1 ± 1.6 kg, -2.2 kg, *p*<0.05).

## Discussion

A major finding of the present study was that whole body fat-free mass in male athletes can be predicted using an equation with body mass-to-waist circumference and waist circumference-to-body height ratios as independent variables. The SEE in this equation (<2.3 kg) is similar to that reported in prior studies, which developed the equation for estimating fat-free mass using anthropometric variables (1.8–2.6 kg) [7]. Thus, the current finding suggests that the accuracy of the prediction equation developed here is comparable to that derived from traditional anthropometric models (body height, circumferences of arm, thigh, calf and skinfold measurements) in spite of the use of the few variables (body height, body mass, and waist circumference).

There was a breakpoint (72.4 kg of BM) in the relationship between measured FFM and BM. In addition, the proportions of whole-body FM and FFM to body mass differed between athletes and untrained individuals [13], as well as among those with habitual training regimens [10–12]. Therefore, we expected that these aspects would influence the possible associations of the adopted anthropometric variables with whole-body FFM and the two sets of dummy variables (athletes vs. sedentary males, and above- vs. below 72.4 kg of BM) used in developing the prediction equation. However, the two dummy variables were not selected as explainable variables for whole-body FFM. These results suggests that the proportions of either whole-body FM or FFM to body mass have less influence in developing an anthropometric model for the prediction of whole-body FFM in athletes.

Furthermore, the proportion of segment FFM to whole-body FFM exhibited event-related differences in this study. Namely, dominant regions of development were FFM of the upper extremity segment in gymnasts, Judo and Kendo athletes, and FFM of the trunk segment in throwers, and FFM of the lower extremity segment in soccer athletes, jumpers, and cyclists. Nevertheless, the developed prediction equation had good validity for predicting whole-body FFM in the cross-validation group. However, it is noteworthy that that the proportion of trunk segment FFM to whole-body FFM was positively correlated to residual error (*r* = 0.476, *p*<0.001) and that the correlation coefficient was higher than those between the ratios of upper and lower extremities FFM and the residual error (*r* = 0.305–0.371, *p*<0.001) but not in FM. In addition, there were significant differences between estimated- and measured FFM in throwers and sedentary males, although these values were smaller when compared to the estimated error in the developed equation (< 2.3 kg). These two groups had a greater ratio of trunk segment FFM to whole body FFM. This finding indicates that the estimated error of the developed prediction equation in this study depends upon the proportion of each segment FFM to whole-body one, but not in FM. In particular, there is a need to pay attention to estimated accuracy when this developed equation is applied to athletes with a greater proportion of trunk segment FFM to whole-body FFM as well as sedentary males.

Multiple regression analysis revealed that BM/W and W/BH were selected as explainable variables for predicting whole body FFM. In this study, BM/W was strongly associated with whole body FFM across all subjects (Table 2). This finding supports previous results showing that this ratio was highly correlated to skeletal muscle volume in children [9]. In this study, waist circumference was strongly correlated with FM in comparison to FFM (Table 2). This finding is consistent with earlier findings that waist circumference is associated with whole body FM [15, 20, 21]. Body mass is the sum of FM and FFM [22]. Taking this aspect into account, Ohta, Midorikawa (9) considered body mass-to-waist ratio to be translated into the following; BM/W~ (FM+FFM)/FM = FM/FM+FFM/FM = 1+FFM/FM, and proposed that the ratio should be higher as FFM within the body increases. On the basis of this idea, these researchers examined how the ratio is associated with total skeletal muscle volume in children and found a very strong correlation between the two variables. Furthermore, many earlier findings indicate that waist circumference-to-body height ratio can be an indicator of fatness [16]. The association between the ratio and FFM is weak in untrained adult males (*r* = 0.17) [23]. In the current results, however, W/BH was strongly correlated to not only fat components (*r* = 0.786–0.883) but also to FFM (*r* = 0.678) in male athletes (Table 2), which supported the selection of W/BH as an explainable variable for estimating FFM.

There are some limitations in the present study. Firstly, location of the waist circumference measurement may influence the accuracy of FFM estimation since mean values of waist circumference differ among locations of waist circumference measurement (the narrowest waist < immediately below the lowest ribs < the midpoint between the lowest rib and the iliac crest < immediately above the iliac crest) whereas any waist circumference values are highly correlated to fat mass [15], whereas any waist circumference measured immediately above the iliac crest should be used for the developed prediction equation in this study. Secondly, we measured only waist circumference in the prediction of whole body FFM. In an earlier finding [7], arm, thigh, and calf circumferences and skinfolds have been adopted as used as an anthropometric model for estimating appendicular FFM. Unfortunately, we did not have the relevant data to allow the comparisons of the estimated accuracy between the current equation and the equation reported in the earlier study. On the other hand, the SEE in the developed equation is comparable to that reported by the earlier finding [7].

In conclusion, the present study indicates that an equation with body mass-to-waist circumference and waist circumference-to-body height as independent variables is applicable for predicting whole-body fat-free mass in male athletes. The estimated accuracy of the developed equation is comparable to that of anthropometric models derived in the prior studies.

## Supporting information

S1 Dataset (XLSX)
